# Copper(II) and Zinc(II) Complexes with the Clinically Used Fluconazole: Comparison of Antifungal Activity and Therapeutic Potential

**DOI:** 10.3390/ph14010024

**Published:** 2020-12-30

**Authors:** Nevena Lj. Stevanović, Ivana Aleksic, Jakob Kljun, Sanja Skaro Bogojevic, Aleksandar Veselinovic, Jasmina Nikodinovic-Runic, Iztok Turel, Miloš I. Djuran, Biljana Đ. Glišić

**Affiliations:** 1 Department of Chemistry, Faculty of Science, University of Kragujevac, R. Domanovića 12, 34000 Kragujevac, Serbia; nevena.stevanovic@pmf.kg.ac.rs; 2 Institute of Molecular Genetics and Genetic Engineering, University of Belgrade, Vojvode Stepe 444a, 11042 Belgrade, Serbia; ivana_aleksic@imgge.bg.ac.rs (I.A.); sanja.bogojevic@imgge.bg.ac.rs (S.S.B.); 3 Faculty of Chemistry and Chemical Technology, University of Ljubljana, Večna pot 113, SI-1000 Ljubljana, Slovenia; Jakob.Kljun@fkkt.uni-lj.si; 4 Department of Chemistry, Faculty of Medicine, University of Niš, Blvd. Dr Zorana Djindjica 81, 18108 Niš, Serbia; aveselinovic@medfak.ni.ac.rs; 5 Department of Chemical and Biological Sciences, Serbian Academy of Sciences and Arts, Knez Mihailova 35, 11000 Belgrade, Serbia

**Keywords:** copper(II) complex, zinc(II) complex, fluconazole, antifungal agents, anti-biofilm activity

## Abstract

Copper(II) and zinc(II) complexes with clinically used antifungal drug fluconazole (fcz), {[CuCl_2_(fcz)_2_]^.^5H_2_O}_n_, **1**, and {[ZnCl_2_(fcz)_2_]·2C_2_H_5_OH}_n_, **2**, were prepared and characterized by spectroscopic and crystallographic methods. The polymeric structure of the complexes comprises four fluconazole molecules monodentately coordinated via the triazole nitrogen and two chlorido ligands. With respect to fluconazole, complex **2** showed significantly higher antifungal activity against *Candida krusei* and *Candida parapsilosis*. All tested compounds reduced the total amount of ergosterol at subinhibitory concentrations, indicating that the mode of activity of fluconazole was retained within the complexes, which was corroborated via molecular docking with cytochrome P450 sterol 14α-demethylase (CYP51) as a target. Electrostatic, steric and internal energy interactions between the complexes and enzyme showed that **2** has higher binding potency to this target. Both complexes showed strong inhibition of *C. albicans* filamentation and biofilm formation at subinhibitory concentrations, with **2** being able to reduce the adherence of *C. albicans* to A549 cells in vitro. Complex **2** was able to reduce pyocyanin production in *Pseudomonas aeruginosa* between 10% and 25% and to inhibit its biofilm formation by 20% in comparison to the untreated control. These results suggest that complex **2** may be further examined in the mixed *Candida-P. aeruginosa* infections.

## 1. Introduction

**Figure 1:**
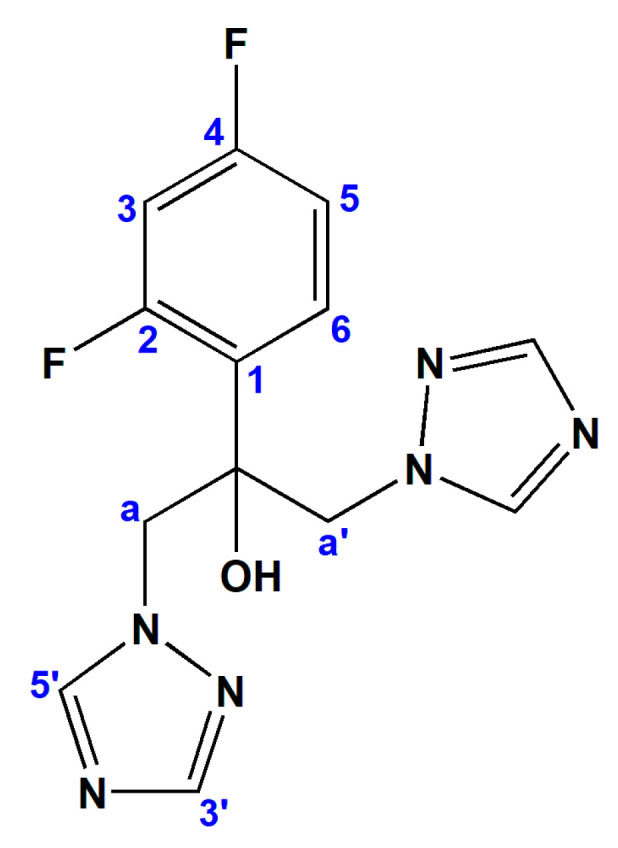
Structural formula of fluconazole (fcz). The numbering of atoms was used for NMR assignation and does not match the one applied in the X-ray study of the complexes **1** and **2**.

Over the last few decades, fungal strains causing invasive infections present not only a serious threat to human health worldwide, but also causing devastating effect for agriculture and the environment [1,2,3]. An estimated 1.5–2 million people die of a fungal infection each year and this mortality is mainly caused by *Aspergillus, Candida, Cryptococcus*, and *Pneumocystis* species [4]. Immunocompromised patients, such as recipients of solid organ transplants or hematopoietic stem cells, and those infected with HIV are most susceptible to these pathogens [5]. Four classes of organic compounds classified based on their mode of action are currently used for the treatment of fungal infections: the polyenes (including amphotericin B and nystatin), azoles, echinocandins (caspofungin and micafungin), and antimetabolites (such as 5-fluorocytosine) [6]. Among them, azoles are the most commonly used antifungal agents, due to the fact that they have broad-spectrum antifungal activity, better effectiveness and an acceptable toxicity profile [7]. This class of antifungal agents inhibits the enzymes (cytochrome P450), included in the synthesis of ergosterol, which is a component of the fungal cell membrane [8,9]. Fluconazole (fcz), which belongs to the first-generation azoles, is a triazole (Figure 1), developed for the treatment of *Candida* infections, including *C. albicans*, *C. tropicalis*, *C. parapsilosis*, and dermatophytes [8]. On the other hand, *Aspergillus* spp. or other hyphomycetes and *C. krusei* are intrinsically resistant to fluconazole, while *C. glabrata*, occasionally shows a reduced sensitivity [8]. Moreover, the number of fluconazole-resistant strains has rapidly increased with the excessive use of this antifungal agent [10]. Considering the limited antifungal spectrum of fluconazole, as well as resistance development and its other drawbacks, the identification of novel compounds capable to overcome these problems is highly urgent. 

**Figure 2:**
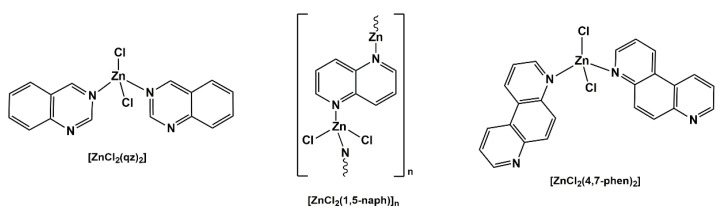
Structural formulas of zinc(II) complexes which showed good anti-*Candida* activity (qz is quinazoline, 1,5-naph is 1,5-naphthyridine and 4,7-phen is 4,7-phenanthroline) [24].

Due to the favorable properties and proven application in medicine [11], metal complexes could represent an untapped source of novel compounds having antifungal potential. It is well known that the presence of metal ions is essential for many biological processes in living organisms, such as metabolism, respiration, photosynthesis, growth and reproduction, muscle contraction and respiration, nerve signal transmission and nitrogen fixation [12]. Without the presence of metal ions, most enzymes would not be able to conduct their impressively fine-tuned transformations [13]. As was mentioned above, besides the essential role of metal ions in biological systems, many metal-based drugs and imaging agents are extensively used in medicine for the treatment and diagnosis of a wide range of diseases [11]. The main advantage of the medicinal use of metal compounds with respect to purely organic drugs is a different mode of action, including ligand exchange or release, reactive oxygen species (ROS) generation, redox activation and catalytic generation of toxic species or depletion of essential substrates, being difficult or even impossible to achieve with an organic compound alone [14]. Moreover, metal complexes are endowed with a vast variety of different geometries and possess a higher three-dimensionality compared to the generally flat organic compounds, which has been connected to their higher clinical success rates [15]. One of the strategies to overcome the antifungal resistance is to combine biologically active or pharmaceutically used organic compounds with metal ions, which have shown antimicrobial properties [16]. It is proposed that chelation reduces the polarity of the metal ion and could enhance its lipophilic character, which subsequently favors the permeation through the lipid layers of the cell membrane and blocks the metal binding sites on enzymes of microorganisms [17]. Up to now, various copper(II) and zinc(II) complexes with nitrogen-donor ligands have been synthesized and shown remarkable antifungal and antibacterial activities and could represent novel agents for potential clinical use [18,19,20,21,22,23,24,25]. Recently, we have synthesized three zinc(II) complexes with aromatic nitrogen-containing heterocycles, [ZnCl_2_(qz)_2_], [ZnCl_2_(1,5-naph)]_n_ and [ZnCl_2_(4,7-phen)_2_] (qz is quinazoline, 1,5-naph is 1,5-naphthyridine and 4,7-phen is 4,7-phenanthroline; Figure 2), which showed good inhibitory activity against two *Candida* strains (*C. albicans* and *C. parapsilosis*), while not inducing toxic effects on the healthy human fibroblast cell line (MRC-5) [24]. Moreover, [ZnCl_2_(4,7-phen)_2_] complex manifested the ability to modulate *Candida* hyphae formation and showed a synergistic effect with clinically used nystatin. Zinc(II) complexes with antifungal agent itraconazole (itraco), [Zn(itraco)_2_Cl_2_]^.^2H_2_O and [Zn(itraco)_2_(OH)_2_]^.^H_2_O, were more active against *Sporothrix brasiliensis* and *Sporothrix schenckii* yeasts than itraconazole, and, additionally, showed significant activity against three clinically important protozoans, *Leishmania amazonensis*, *Trypanosoma cruzi* and *Toxoplasma gondii* [25]. Fluconazole was also used as a ligand for the synthesis of different copper(II) and zinc(II) complexes [26,27,28,29,30,31,32,33,34,35].

Considering all the above-mentioned facts, in the present study, we report the synthesis and structural characterization of new copper(II) and zinc(II) complexes with clinically used antifungal drug fluconazole, {[CuCl_2_(fcz)_2_]^.^5H_2_O}_n_, **1**, and {[ZnCl_2_(fcz)_2_]·2C_2_H_5_OH}_n_, **2**. These two complexes and fluconazole were evaluated for the in vitro antimicrobial potential, which was compared to the cytotoxicity toward the normal human fibroblast cell line (MRC-5) with the aim to determine their safety profile. The effect of the complexes and fluconazole on the amount of ergosterol, fungal biofilm formation and filamentation of *C. albicans* has been investigated in order to obtain an insight into the mode of action of the synthesized fluconazole-metal complexes.

## 2. Results and Discussion

### 2.1. Synthesis and Structural Characterization of Complexes ***1*** and ***2***

**Figure 3:**
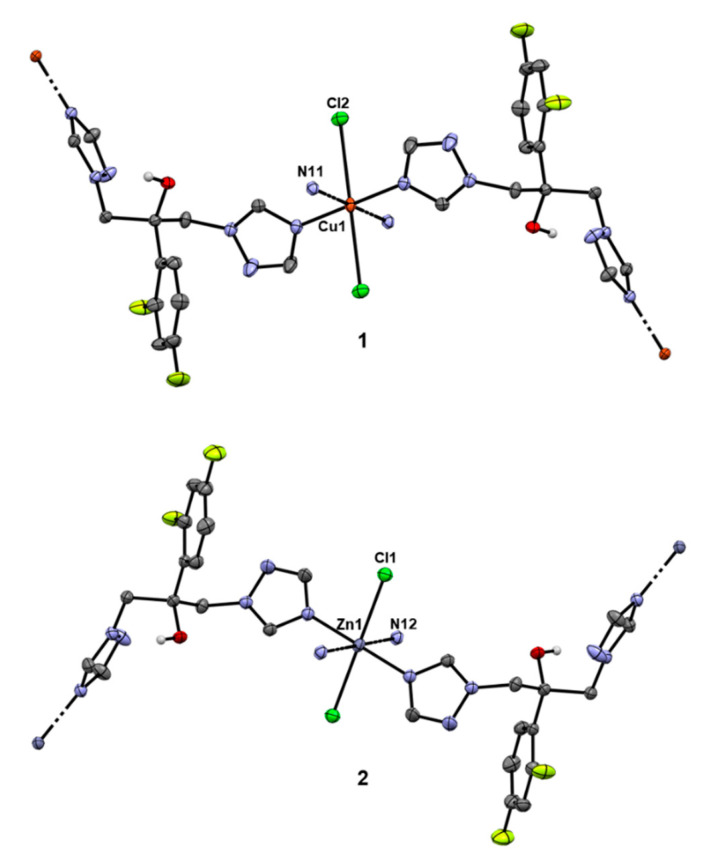
Crystal structures of complexes **1** and **2**. Thermal ellipsoids are presented at a 35% probability level. Solvent molecules are omitted and only hydrogen atoms on the oxygen atoms of fluconazole ligands are displayed for better clarity of presentation.

Antifungal agent, fluconazole (fcz), was used as a ligand for complexation to Cu(II) and Zn(II) ions and the corresponding complexes, {[CuCl_2_(fcz)_2_]^.^5H_2_O}_n_, **1**, and {[ZnCl_2_(fcz)_2_]·2C_2_H_5_OH}_n_, **2**, were isolated as final products (Figure 3). The procedure given in the Materials and Methods section was applied for the synthesis of these two complexes (vide infra). The crystals of the copper(II) complex **1** suitable for X-ray diffraction analysis were obtained after the blue precipitate from the reaction was recrystallized in the mixture of acetonitrile/water (*v*/*v* 1:1) after standing at room temperature for 3 to 5 days, while those of zinc(II) complex **2** were obtained by slow evaporation of the mother ethanolic solution at room temperature. The structure of the complexes was confirmed by mass spectrometry, IR and UV-Vis spectroscopy and single-crystal X-ray diffraction analysis, while complex **2** was additionally characterized by ^1^H and ^19^F NMR spectroscopy.

#### 2.1.1. Solid State Studies

Complexes **1** and **2** are isostructural, both crystallizing in monoclinic space group C2/c. The fluconazole ligand acts as a bridging ligand through both triazole rings, which considering the metal-to-ligand ratio 1:2 results in octahedral geometry of the complexes with N_4_Cl_2_ metal coordination spheres (Figure 3; overlay of the metal coordination spheres in **1** and **2** is displayed in Supplementary Figure S1). In solid state, the complexes present polymeric interlinked structures. The octahedral environment in the copper(II) complex is slightly distorted due to the electronic (Jahn–Teller) effect of the Cu(II) electron sphere, which can be observed through the increase of the M–Cl bond length from 2.553 to 2.802 Å (Supplementary Table S1). The M–N distances show a minimal difference, whereas, in the case of copper(II) complex **1**, the distance is 0.1 Å shorter (2.006 and 2.037 Å for **1** vs. 2.130 and 2.167 Å for **2**; Supplementary Table S1). The structures also show a large cavity occupied by five water molecules in the case of **1**, which form a hydrogen bond network including two hydroxyl groups of separate fcz ligands, as well as weaker interactions with coordinated chlorido ligands (Supplementary Figure S2). In the case of **2**, the five water molecules are replaced by two ethanol molecules each forming separate hydrogen bonds with respective OH groups and interacting with coordinated chlorido ligands (Supplementary Figure S3). Supplementary Table S2 contains additional information on the structural data, data collection and structure refinement.

The same coordination mode of fluconazole was observed in the previously reported copper(II) and zinc(II) complexes having fluconazole moiety in their structures [26,27,28,29,30,31,32,33,34,35], while the difference in the structure of the complexes **1** and **2** and those previously reported originates from the geometry and nuclearity, as well as from the type of auxiliary ligands or solvate molecules.

The IR spectra of the complexes **1** and **2**, recorded in the range of 4000–400 cm^−1^, show the expected bands attributable to the coordinated fluconazole [36] and crystallization water and ethanol molecules, respectively. Thus, both complexes exhibited a broad absorption at ~3300 cm^−1^, which was attributed to the O–H stretching vibrations of fluconazole, as well as crystallization water and ethanol molecules [36]. The shifting of characteristic vibrations due to the aromatic rings, ν(C_ar_=C_ar_) and ν(C_ar_=N), for complexes **1** and **2** in respect to those of free fluconazole, observed in the region 1620–1370 cm^−1^, confirms the coordination of this antifungal agent to Cu(II) and Zn(II) ions.

#### 2.1.2. Solution Behavior of Complexes **1** and **2**

The molar conductance data for the complexes **1** and **2**, given in the Materials and Methods section, are in accordance with their non-electrolytic nature in DMSO, as a solvent used for the preparation of a stock solution for biological evaluation of the complexes [37,38]. Moreover, the values of the molar conductance measured immediately after the dissolution of the complexes did not change significantly during 48 h, indicating their stability during this time. 

The UV-Vis spectra of complexes **1** and **2** were recorded in DMSO at room temperature. As can be expected, the copper(II) complex **1** exhibits a single broad band at 879 nm [39], assigned to the *d*_z_^2^, *d*_xy_, *d*_xz_, *d*_yz_→*d*_x_^2^_-y_^2^ transitions with a *d*_x_^2^_−y_^2^ ground state [40]. On the other hand, the absorbance peak at 261 nm, observed in the UV-Vis spectrum of zinc(II) complex **2**, is associated with the characteristic intraligand charge transfer transitions [24]. With the aim to follow solution stability of the complexes during the time, **1** and **2** were dissolved in DMSO, and their UV-Vis spectra were recorded immediately after dissolution, and after 24 and 48 h of standing at room temperature (as an illustration, time-dependent UV-Vis spectra of complex **1** were shown in Supplementary Figure S4a). The shape of spectra, intensity and position of the absorption maxima of **1** and **2** remained unmodified during the investigated time, being in accordance with the stability of these complexes in solution.

The positive ion ESI mass spectra for **1** and **2** show peaks at *m*/*z* = 675.1372 and 711.1059, which are consistent with theoretical *m*/*z* values calculated for [Cu(fcz)_2_]^2+^ and [ZnCl(fcz)_2_]^+^ cations, respectively. In both cases, the isotopic pattern is in accordance with that simulated for the corresponding molecular cation, confirming its composition. 

The ^1^H and ^19^F NMR spectra of zinc(II) complex **2** were recorded in acetone-*d*_6_ and compared with those for the uncoordinated fluconazole. Both these spectra were assigned based on the previous assignments of the corresponding spectra of fluconazole recorded in DMSO-*d*_6_ [36]. All proton signals of the complex **2**, except that for C3H proton, are shifted downfield in respect to those of the uncoordinated fluconazole. The chemical shifts of fluconazole protons after its coordination to the Zn(II) ion are found to be strongly dependent on the proton distance from the metal ion. The largest shifts were observed for the protons, which are adjacent to the N4 triazole nitrogen binding atom, i.e., C5′H and C3′H protons. Thus, these two protons in the spectrum of fluconazole give signals at *δ* 8.29 and 7.76 ppm, which were shifted downfield at 8.53 (Δ*δ* = +0.24 ppm) and 7.91 (Δ*δ* = +0.15 ppm) ppm, respectively, after the formation of the complex **2**. The presence of ethanol in the structure of complex **2** can be also confirmed by the singlet at *δ* 3.39 ppm due to the hydroxyl proton, and the signals at 1.12 and 3.57 ppm due to the methyl and methylene ethanol protons, respectively. From the time-dependent NMR measurements performed in DMSO-*d*_6_/D_2_O (*v*/*v* 3:1) (Supplementary Figure S4b), it can be concluded that fluconazole remains coordinated to the Zn(II) ion during 48 h and that no coordination of DMSO was observed during this time.

### 2.2. Comparative Biological Activity Evaluation of Fluconazole and Complexes ***1*** and ***2***

#### 2.2.1. In Vitro Anti-Candida Properties

Fluconazole has been reported to be effective in treating candidiasis in the late 80s [41] with more and more strains appearing to show the resistance to this agent, so further modification of its structure is a good strategy to find a compound with perspective to become a new antifungal drug. Complexation with metal ions that were shown to induce response in microorganisms, especially fungi [24,42,43], was undertaken in this study and the results of the antifungal efficiency in terms of growth inhibition are presented in Table 1.

As can be seen from Table 1, in most cases, the complexation of fluconazole with Zn(II) ion improved the minimal inhibitory concentration (MIC) values in comparison to those of fluconazole. Thus, the 11- and 5.4-fold improvement was observed in the case of *C. krusei* and *C. parapsilosis*, respectively, for complex **2** (µM concentrations; Table 1). Both complexes showed the higher activity (2.7-fold improvement; µM) than fluconazole against *C. albicans* clinical isolates, and the activity decreased in the case of Cu(II) complex **1** against *C. albicans* and *C. krusei* type of strains in comparison to that of fluconazole. These findings are in line with the recent study of Guo et al. [44], who reported a new polyoxovanadate functionalized by zinc(II)-fluconazole complex with good activity on several clinical *Candida* strains. Notably, the complexation of fluconazole with the Cu(II) and Zn(II) ions resulted in the 11.4- to 12.7-fold increased cytotoxicity, however the selectivity indices of each complex were up to 41 and 73 for **1** and **2**, respectively (Table 1).

**Figure 4:**
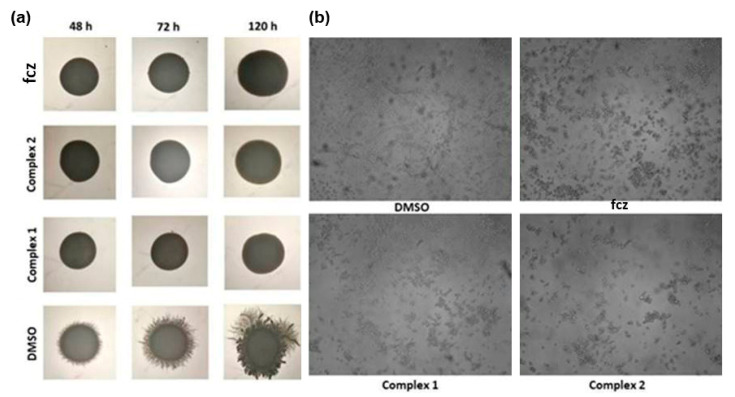
Filamentation of *C. albicans* ATCC 10231 in the presence of subinhibitory (0.5 × MIC value) concentrations of Figure 1. and **2** on the solid Spider medium (**a**) and in RPMI broth (**b**) (Olympus BX51, Applied Imaging Corp., San Jose, CA, United States, under 20× magnification).

One of the main pathogenic properties of *Candida* spp. is the morphological transformation from yeast-to-hyphae form. In recent studies, the presence of metal ions, such as Ag(I) and Ni(II), as well as Mn(II) and Cd(II), was shown to inhibit cellular differentiation of *Candida* [45,46]. Therefore, the effects of complexes **1** and **2**, together with fluconazole, at 0.5 × MIC concentrations, on *C. albicans* ATCC 10231 hyphae formation both on hyphae promoting solid Spider medium and in RPMI (Roswell Park Memorial Institute) broth, were analyzed using light microscopy (Figure 4). The treatment with complexes **1** and **2** and fluconazole provided strong filamentation inhibition in comparison to the DMSO control. Complexes were able to completely prevent hyphae formation on the solid medium (Figure 4a), as well as in broth (Figure 4b), to a comparable level with fluconazole. These results are in line with the previous study showing that pyrazole ligands and metal complexes significantly reduced the yeast-to-hyphae transition of *C. albicans* (inhibition ranging from 30% to 54%) [47]. 

**Figure 5:**
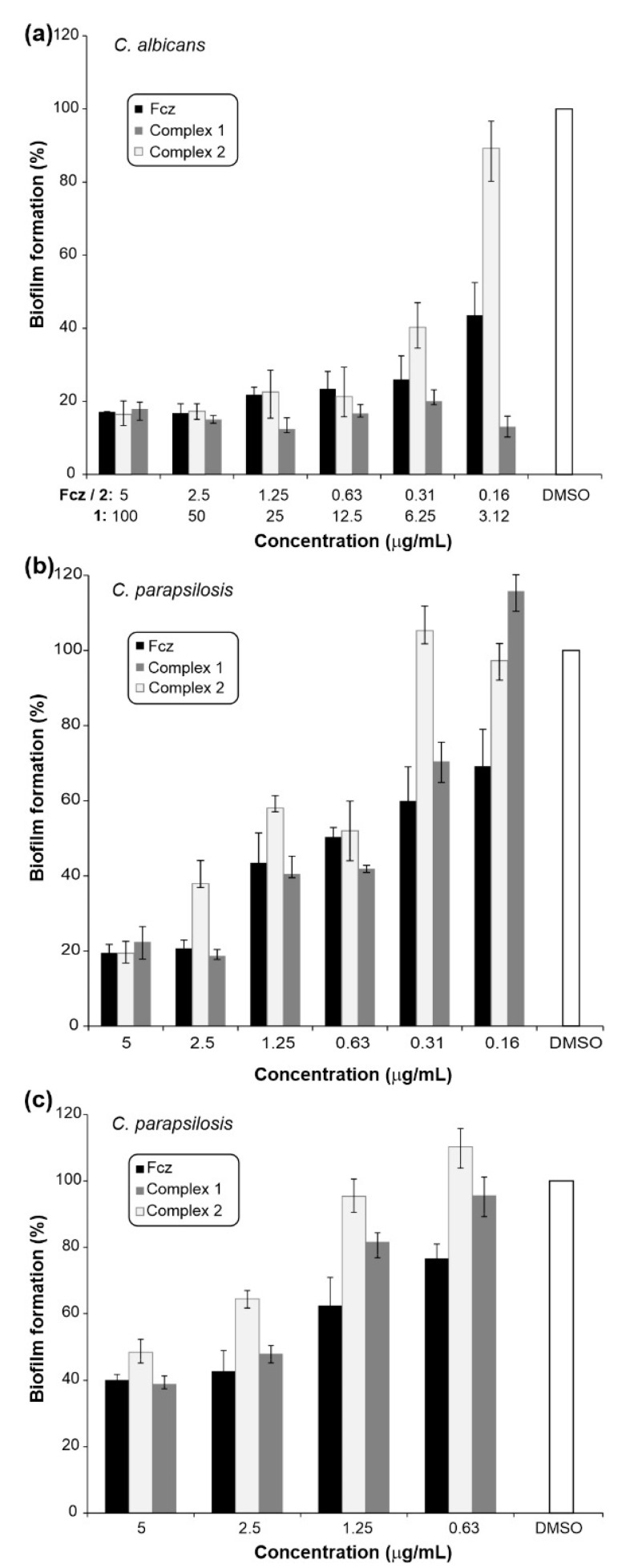
The effect of fluconazole and complexes **1** and **2** on the biofilm formation and eradication. Percentage of formed biofilm of *C. albicans* ATCC 10231 (**a**) and *C. parapsilosis* ATCC 22019 (**b**) in the presence of complexes **1** and **2** and fluconazole and remaining *C. parapsilosis* biofilms treated with complexes **1** and **2** and fluconazole (**c**).

Yeast-to-hyphae transition is an important process during the infection process, and it is the early step in *Candida* biofilm formation. Given that high level of resistance is associated with the biofilms, it is of great importance to evaluate antibiofilm activity of novel antifungals [48]. Hence, the comparative activity of fluconazole and its complexes **1** and **2** were assessed both on biofilm formation and eradication (Figure 5). Complexes **1** and **2** showed good to moderate effect on biofilm formation of *C. albicans* ATCC 10231 and *C. parapsilosis* ATCC 22019 strains (Figure 5a,b) and were able to disperse pre-formed biofilm of *C. parapsilosis* ATCC 22019 (Figure 5c). Overall, copper(II) complex **1** showed slightly higher efficiency in the anti-biofilm activity. The highest inhibition percentage was 87.6% for complex **1** tested on biofilm formation of *C. albicans* in a concentration of 25 µg/mL and the comparable effect was reached at 3.12 µg/mL (Figure 5a). In the case of *C. parapsilosis* biofilms, the good concentration gradient between 5–0.63 µg/mL for complex **1** is notable (Figure 5b). In biofilm eradication assays, good efficacy was noted on *C. parapsilosis* pre-formed biofilms in concentrations 5 µg/mL and 2.5 µg/mL, with the effect comparable to that of fluconazole (Figure 5c). Indeed, the anti-biofilm activity of fluconazole has been previously reported on *C. glabrata, C. parapsilosis* and *C. rugosa* biofilms, with fluconazole causing significant damage to the vitality and integrity of *Candida* cells [49]. In addition, Gomes da Silva Dantas et al. demonstrating the inhibition of 9.9% of the *C. glabrata and C. krusei* biofilms by copper(II) complex with 2-thiouracil [50].

**Figure 6:**
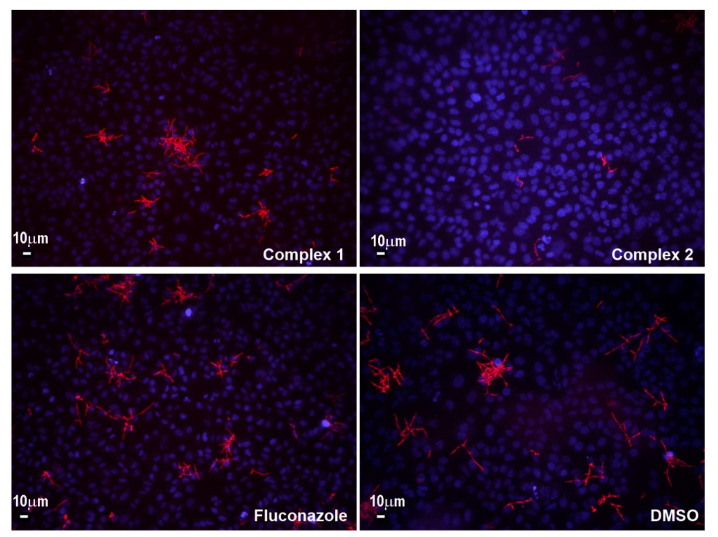
The adhesion of fluorescent *C. albicans* SC5314-RFP on A549 cells in the presence of complexes **1** and **2**, and fluconazole (Olympus BX51, Applied Imaging Corp., San Jose, CA, United States, under 20× magnification; scale bar representing 10 µm). DAPI (2-(4-amidinophenyl)-6-indolecarbamidine dihydrochloride) stained nuclei appear in blue, while fluorescent red is from labeled *C. albicans* cells.

To study the effect of complexes **1** and **2** and fluconazole on *Candida* pathogenesis more thoroughly, the effect on fluorescent *Candida* species adherence to A549 cells, which is an initial step of the invasion of host cells, was analyzed in the presence of the complexes (Figure 6). In both fluorescent *C. albicans* sp. (*C. albicans* SC5314-RFP (RFP is red fluorescent protein) and *C. albicans* SC5314-GFP (GFP is green fluorescent protein)), zinc(II) complex **2** is reducing the adhesion of fungal cells, while fluconazole and copper(II) complex **1** are not showing that effect under the tested conditions. The adhesion of fluorescent *C. albicans* SC5314-RFP on A549 cells in the presence of complexes **1** and **2**, and fluconazole is presented in Figure 6. Sensing and homeostasis of both Cu(II) and Zn(II) ions are crucial in *Candida* differentiation and pathogenesis, and number of genes and proteins have been described to be involved [51,52,53]. Therefore, complexes based on antifungal drugs and these metal ions may have additional targets amongst these genes, proteins they encode, or their regulators.

#### 2.2.2. Ergosterol Biosynthesis in C*. albicans* Treated with Fluconazole and Complexes **1** and **2**

**Figure 7:**
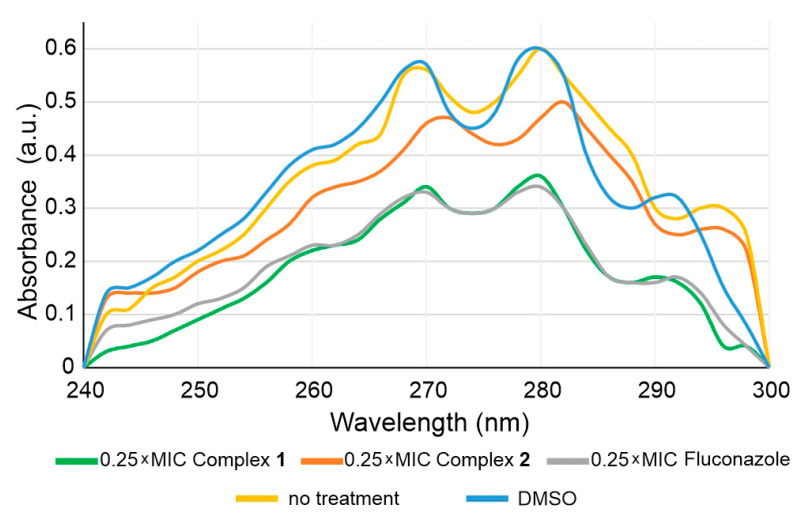
UV spectrophotometric ergosterol profiles between 240 nm and 300 nm of *C. albicans* cultures treated with 0.5 × MIC concentrations of complexes **1** and **2** and fluconazole.

Ergosterol is a sterol found in the fungal cell membrane possessing similar functions like cholesterol in animal cells, which regulates cell membrane permeability and fluidity [54,55]. Ergosterol biosynthesis, more specifically interruption of the conversion of lanosterol to ergosterol via binding to fungal cytochrome P450, is a mechanism of action of azole drugs, including fluconazole [56,57]. Therefore, the total amount of ergosterol in *C. albicans* treated with subinhibitory concentrations of complexes **1** and **2** in comparison to fluconazole was determined (Figure 7).

All three compounds reduced the total amount of ergosterol upon treatment with 0.5 × MIC concentrations, with complex **1** being the most potent, while complex **2** and fluconazole showed a comparable effect. Based on this, the mode of activity could be similar to the one of fluconazole itself, while it is possible that complex **1** is interacting with some additional targets to cause even further decrease in the total amount of ergosterol. These findings are in line with previous results published by Guo et al., which represented the same effect of polyoxovanadate functionalized by zinc(II)-fluconazole complex [44]. They studied the mechanism of ergosterol biosynthesis reduction by the latter complex by evaluating the expression of five genes involved in ergosterol biosynthesis (ERG1, ERG7, ERG11, ERG27 and ERG28) and showed that this complex may also inhibit the ERG gene expression.

**Figure 8:**
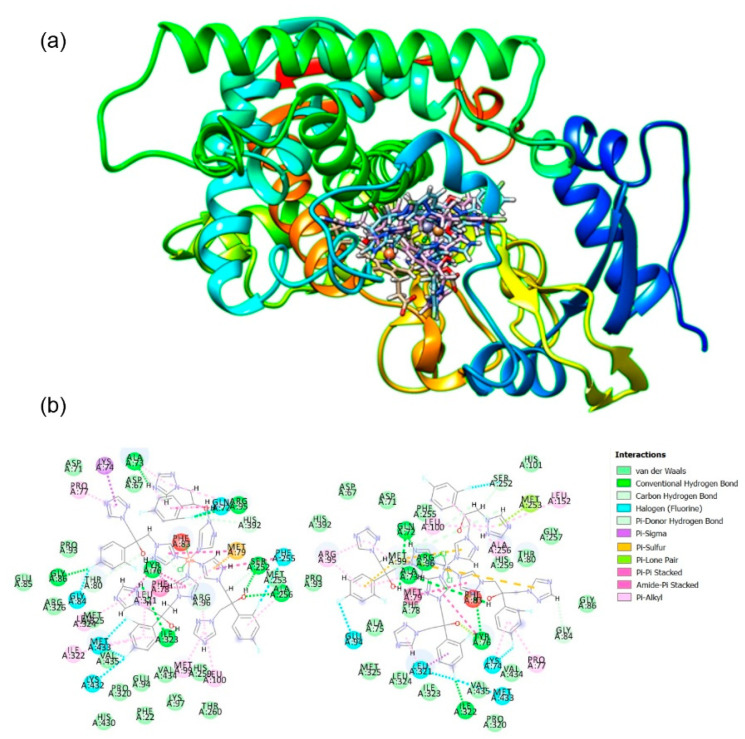
Molecular docking of complexes **1** and **2** and CYP51. (**a**) Best calculated poses for the complexes **1** and **2** inside the active site of CYP51 (PDB 1EA1); (**b**) Two-dimensional representation of calculated interactions between complex **1** (**left**) and complex **2** (**right**) and amino acids from the enzyme active site.

Microbial CYPs are targets of drugs and agrochemicals and, in particular, CYP51 is exhibiting evolution to resistance in the clinic and the field [9]. According to the molecular docking results, both complexes **1** and **2** show binding preferences near to cofactor of fungal CYP51 (Figure 8). 

Interaction of antifungal azoles with CYP51 has been studied in detail and it was postulated that triazoles form van der Waals contact within hydrophobic pocket of the active site [58]. If in metal complexes, azole nitrogen atoms are involved in coordination, binding to iron in heme is prevented. It seems reasonable that such complexes thus inhibit the fungal growth via other mechanisms of action, as was already proposed in the case of antifungal activity of ruthenium(II) complexes [59]. 

The investigated complexes **1** and **2** show steric, hydrophobic, H-bonding and non-planar interactions with amino acids of the binding site, but no direct metal CYP51 bond was found. The main mechanism of binding stabilization is related to the electrostatic and van der Waals interactions with protein backbone, so insertion between the stacking amino acids and complex with extended fused planar aromatic system can lead to the intercalative binding. Both complexes **1** and **2** have sufficient electrostatic and π stacking interactions with amino acids, as well as hydrogen bonding interactions. Molecular docking assessment also revealed van der Walls interactions (VdW) to be higher for the complex **1** (−1951.52 kcal/mol) in comparison to complex **2** (−1371.7 kcal/mol). The HBond score function estimates hydrogen bonding ability and it was −4.05955 and −13.8199 kcal/mol for **1** and **2**, respectively. Complex **2** had higher electrostatic interaction (−42.334 kcal/mol) in comparison to complex **1** (−26.85 kcal/mol). Steric interactions are quantified with score function Steric, and according to the obtained results, complex **2** had higher interaction (−137.293 kcal/mol) in comparison to complex **1** (−87.6611 kcal/mol). Complex **2** also had higher internal energy interactions (−60.2078 kcal/mol) in comparison to complex **1** (−52.1336 kcal/mol). Moldock score identified complex **2** to have higher binding potency in comparison to complex **1** (obtained values for Moldock score were −101.668 and −34.154 kcal/mol, respectively).

#### 2.2.3. Antibacterial and Anti-QS Effects

There is a general trend in drug repurposing efforts to examine existing non-antibiotic drugs as the quorum sensing inhibitors [60]. Given that copper(II) complexes with aromatic nitrogen-containing ligands were shown to effectively interfere with bacterial cell-to-cell signalling [61], complexes **1** and **2** along with fluconazole were also examined in terms of antibacterial and anti-QS activity. Firstly, these complexes did not show any antibacterial activity on four *Pseudomonas aeruginosa* strains, two *Staphylococcus aureus* strains, and *Escherichia coli*. MIC values on all tested strains were above 200 µg/mL, and that was not considered as significant activity (data not shown). On the other side, complex **2** was able to reduce the pyocyanin production between 10% and 25% in three clinical isolates of *P. aeruginosa* (Table 2), while complex **1** and fluconazole were not able to interfere with this virulence factor. Furthermore, complex **2** showed the moderate potency to inhibit biofilm formation of *P. aeruginosa* (Table 2). 

Pyocyanin production and biofilm formation in *P. aeruginosa* are virulence factors regulated trough the quorum sensing. Quorum sensing network in *P. aeruginosa* has been studied in depth and it is known that pyocyanin production and biofilm formation is regulated trough the PQS signaling pathway [62]. For clarification of the mode of observed activity, specific biosensors were employed and the PQS pathway for complex **2** was confirmed (Supplementary Table S3).

## 3. Materials and Methods

### 3.1. Materials and Measurements

Metal(II) salts, CuCl_2_^.^2H_2_O and ZnCl_2_, fluconazole, ethanol, acetonitrile, dimethyl sulfoxide (DMSO), deuterated dimethyl sulfoxide (DMSO-*d*_6_), deuterium oxide (D_2_O) and deuteroacetone (acetone-*d*_6_) were purchased from commercial suppliers (Sigma-Aldrich Chemical Co, Munich, Germany, and Acros Organics, Geel, Belgium). All chemicals were of reagent-grade quality or higher and used as received. 

Elemental microanalysis measurements of the complexes **1** and **2** were done using a PerkinElmer 2400 Series II instrument (CHN). The ESI-HRMS spectra in the positive mode of the complexes dissolved in acetonitrile were recorded with an Agilent 62224 accurate mass spectrometer. Infrared spectra were recorded on a Bruker FTIR Alpha Platinum ATR spectrometer in the wavenumber range 4000–400 cm^−1^. UV-Vis spectra were collected on a Perkin-Elmer Lambda 750 UV/Vis/near-IR spectrophotometer after dissolving the corresponding complex in DMSO. The concentration of the solutions used for these measurements was 1 × 10^−2^ and 5 × 10^−4^ M for complexes **1** and **2**, respectively. UV-Vis stability measurements were carried out on a Shimadzu double-beam spectrophotometer after dissolving the corresponding complexes in DMSO immediately after their dissolution and after 24 h and 48 h. The measurements were performed over the wavelength range 1100–200 nm, with the concentration of the solutions being 4 × 10^−3^ M (**1**) and 5 × 10^−4^ M (**2**). The ^1^H and ^19^F NMR spectra of complex **2** were recorded on a Bruker Avance III 500 spectrometer at room temperature using tetramethylsilane as the internal standard. 5.0 mg of fluconazole and complex **2** were dissolved in 0.6 mL of acetone-*d*_6_ and transferred into a 5 mm NMR tube. Chemical shifts, *δ*, and scalar couplings, *J*, are reported in ppm (parts per million) and Hz (Hertz), respectively. The splittings of protons are designated as: *s*, singlet; *dd*, doublet of doublets; *td*, triplet of doublets; *ddd*, doublet of doublet of doublets, *qt*, quartet of triplets; *m*, multiplet. With the aim to check the solution stability of the synthesized zinc(II) complex **2**, ^1^H NMR spectra were recorded immediately after its dissolution and after 2, 24 and 48 h standing at room temperature in DMSO-*d*_6_/D_2_O (*v*/*v* 3:1). Molar conductivity measurements were performed at ambient temperature using digital conductivity-meter Crison Multimeter MM 41. The concentration of the solution of complexes **1** and **2** in DMSO used for these measurements was 1 × 10^−3^ M.

### 3.2. Synthesis of Complexes ***1*** and ***2***

The solution of 1.0 mmol of CuCl_2_^.^2H_2_O (170.5 mg, for **1**) or ZnCl_2_ (136.3 mg, for **2**) in 5 mL of ethanol was added slowly under stirring to the solution of 2.0 mmol of fluconazole (612.5 mg) in 10 mL of ethanol. The obtained solution was stirred at room temperature for 3–4 h and then left to slowly evaporate. The blue precipitate formed in the reaction between CuCl_2_^.^2H_2_O and fluconazole was filtered off and dissolved in 20 mL of acetonitrile/water (*v/v* 1:1). After standing of the obtained solution at room temperature for 3–5 days, the blue crystals of **1** were formed, filtered off and air-dried. On the other hand, crystals of complex **2** were obtained after slow evaporation of the mother ethanolic solution at room temperature. These crystals were filtered off and dried at room temperature. Yield (calculated on the basis of fluconazole): 562.5 mg (67.2%) for **1** and 542.4 mg (64.5%) for **2**.

Anal. calcd for **1** **=** C_26_H_34_Cl_2_CuF_4_N_12_O_7_ (MW = 837.09): C, 37.31; H, 4.09; N, 20.08. Found: C, 37.52; H, 4.01; N, 20.21%. ESI-HRMS (CH_3_CN): *m/z* calcd for [Cu(fcz)_2_]^2+^: 675.1377; found: 675.1372. IR (ATR, *ν*, cm^−1^) ~3300 br (*ν*(O–H)), 3143 w, ~3000 w (*ν*(C_triazole_–H) and (*ν*(C_ar_–H)), ~2950 w(*ν*(C–H)), 1614 m, 1523 w, 1500 m, 1414 m, 1371 m (*ν*(C_ar_=C_ar_) and *ν*(C_ar_=N)), 1439 w (*δ*(CH_2_)), 1271 m (*δ*(O–H)), 1231 m, 1217 m, 1204 m (*β*(C_ar_–H) and *β*(C_triazole_–H)), 1144 m (*ν*(C–N)), 1119vs (*ν*(C–F)), 1084 m (*ν*(C–O)), 1018 m (*β*(C_ar_–H)), 968 m (*β*(C_triazole_–H)), 916 m (*δ*(C–N)), 869 m, 826 w (*γ*(C_ar_–H)), 789 w (*γ*(C_triazole_–H)), 680 s (*γ*(C_ar_–H)), 647 s (triazole ring def.), 579 m (*β*(C_ar_–F)), 527 s (ring def.). UV-Vis (DMSO, *λ*_max_, nm): 879 (*ε* = 99.96 M^−1^ cm^−1^). *Λ*_M_ (DMSO): 7.4 Ω^−1^cm^2^mol^−1^.

Anal. Calcd. for **2 =** C_30_H_36_Cl_2_F_4_N_12_O_4_Zn (MW = 840.98): C, 42.85; H, 4.31; N, 19.99. Found: C, 42.82; H, 4.25; N, 19.76%. ESI-HRMS (CH_3_CN): *m/z* calcd for [ZnCl(fcz)_2_]^+^: 711.1061; found: 711.1059. IR (ATR, *ν*, cm^−1^): ~3200 br (*ν*(O–H)), 3158 w, 3059 w (*ν*(C_triazole_–H) and *ν*(C_ar_–H)), 2976 w (*ν*(C–H)), 1616 m, 1518 m, 1499 m, 1415 m (*ν*(C_ar_=C_ar_) and *ν*(C_ar_=N)), 1483 w (*δ*(CH_2_)), 1271 m (*δ*(O–H)), 1233 w, 1199 m (*β*(C_ar_–H) and *β*(C_triazole_–H)), 1143 m (*ν*(C–N)), 1115 vs (*ν*(C–F)), 1084 m (*ν*(C–O)), 1018 m (*β*(C_ar_–H)), 966 s (*β*(C_triazole_–H)), 913 m (*δ*(C–N)), 865 m, 828 w (*γ*(C_ar_–H)), 788 w (*γ*(C_triazole_–H)), 680 s (*γ*(C_ar_–H)), 650 m (triazole ring def.), 578 m (*β*(C_ar_–F)), 525 m (ring def.). ^1^H NMR (500 MHz, acetone-*d*_6_): *δ* = 8.53 (*s*, 2H, 2C5′H), 7.91 (*s*, 2H, 2C3′H), 7.31 (*td*, *J* = 9.0, 6.7 Hz, 1H, C3H), 7.08 (*ddd*, *J* =11.8, 2.5 Hz, 1H, C6H), 6.86 (*td*, *J* =8.5, 2.6Hz 1H, C5H), 5.77 (*s*, 1H, OH, fcz), 5.00 and 4.83 (*d*, *J* = 14.6 Hz, 4H, CH_2_ a and a′), 3.57 (*qt*, *J* = 7.4, 3.7 Hz, 2H, CH_2_ ethanol), 3.39 (*s*, 1H, OH ethanol), 1.12 (*td*, *J* = 7.0, 0.9 Hz, 3H, CH_3_ ethanol). ^19^F NMR (471 MHz, acetone-*d*_6_): *δ* = −108.68 (*dd*, *J* = 20.5, 8.7 Hz, C2F), −111.64 (*m*, C4F). UV-Vis (DMSO, *λ*_max_, nm): 261 (*ε* = 2.0^.^10^3^ M^−1^ cm^−1^), 267 shoulder (*ε* = 1.7^.^10^3^ M^−1^ cm^−1^). *Λ*_M_ (DMSO): 3.10 Ω^−1^cm^2^mol^−1^.

Abbreviations for the infrared data: br, broad; w, weak; m, medium; s, strong; vs, very strong. 

For comparative purposes, the corresponding data for fluconazole were given in Supplementary Materials.

### 3.3. Crystallographic Data Collection and Refinement of the Structures

For X-ray structural analysis, single crystals of complexes **1** and **2** were surrounded with silicon grease, mounted onto the tip of glass fibres and transferred to the goniometer head in the liquid nitrogen cryostream (150(2) K). Data were collected on a SuperNova diffractometer equipped with Atlas detector using CrysAlis software with monochromated Mo Kα (0.71073 Å) [63]. The initial structural models were obtained via direct methods using the Olex2 graphical user interface [64] implemented in SHELXT. A full-matrix least-squares refinement on *F*^2^ magnitudes with anisotropic displacement parameters for all non-hydrogen atoms using Olex2 or SHELXL-2018/3 was employed [64,65]. All non-hydrogen atoms were refined anisotropically, while the hydrogen atoms were placed at calculated positions and further treated as riding on their parent atoms. The coordinates of water hydrogens were obtained from difference Fourier maps and were further refined using appropriate distance restraints. Additional details on the crystal data, data collection and refinement are given in the Supplementary Table S2. Figures depicting the structures were prepared with Mercury [66].

CCDC 2042947 and 2042948 contain the supplementary crystallographic data. These data can be obtained free of charge from The Cambridge Crystallographic Data Centre via www.ccdc.cam.ac.uk/data_request/cif.

### 3.4. Anti-Candida Properties of Fluconazole and Complexes ***1*** and ***2***

#### 3.4.1. Minimal Inhibitory Concentration (MIC) Values

MIC values of complexes **1** and **2** and fluconazole were determined according to the standard broth micro-dilution assays, in accordance with the Standards of European Committee on Antimicrobial Susceptibility Testing (v 7.3.1: Method for the determination of broth dilution minimum inhibitory concentrations of antifungal agents for yeasts) for *Candida* spp. The tested compounds were dissolved in DMSO at concentration of 50 mg/mL. The highest concentration used was 200 µg/mL. *Candida* strains used in this study included: *Candida albicans* ATCC 10231, *C. parapsilosis* ATCC 22019, *C. krusei* ATCC 6258, and clinical isolates from veterinary and human samples *C. albicans* 1c, *C. albicans* 1f [67], *C. albicans* 11, *C. albicans* 13 [68] and recombinant *C. albicans* SC5314 RFP and *C. albicans* SC5314 GFP [69]. For the MIC assessment, the inoculums were 1 × 10^5^ colony forming units (cfu)/mL. The MIC value was recorded as the lowest concentration that inhibited the growth after 24 h at 37 °C, using the Tecan Infinite 200 Pro multiplate reader (Tecan Group Ltd., Männedorf, Switzerland). 

#### 3.4.2. Anti-Biofilm Activity Assessment

The effect on biofilms formation and eradication of pre-formed biofilms was determined for two selected *Candida* strains (*C. albicans* ATCC 10231 and *C. parapsilosis* ATCC 22019). Anti-biofilm assays were conducted using a previously reported methodology [67,70] with some modifications. In the biofilm inhibition and biofilm eradication assays, inoculums were 1 × 10^6^ cfu/mL. Starting concentrations of the complexes were 5 μg/mL with two-fold serial dilutions following, except for complex **1** on *C. albicans* ATCC 10231, which was 100 μg/mL. The lowest concentration that inhibited biofilm formation was evaluated after incubation for 48 h at 37 °C. In biofilm eradication assays, pre-formed biofilms (24 h at 37 °C) were incubated for 24 h with decreasing concentrations of complexes and fluconazole. Biofilm growth was quantified by crystal violet (CV) staining of adherent cells and estimated as absorbance at 530 nm on Tecan Infinite 200 Pro multiplate reader (Tecan Group Ltd., Männedorf, Switzerland).

#### 3.4.3. Yeast-to-Hyphae Transition

The effect of complexes **1** and **2**, and fluconazole on *C. albicans* ATCC 10231 hyphae formation was assessed using solid Spider medium and RPMI (Roswell Park Memorial Institute Medium) broth. Morphological changes of *C. albicans* ATCC 10231 in the presence and absence of complexes **1** and **2** and fluconazole in subinhibitory concentration (0.5 × MIC value) were analyzed upon *C. albicans* ATCC 10231 growth on Spider medium, as previously described [71]. Hyphae formation was followed in RPMI broth, as well. *C. albicans* cells from an overnight culture grown in Sabouraud dextrose broth medium at 30 °C were washed with PBS (phosphate-buffered saline) and diluted in RPMI 1640 medium with 2% (*v/w*) glucose to the final concentration of 1 × 10^6^ cells/mL. Cell suspension was treated with 0.5 × MIC concentrations of both complexes and fluconazole for 3 h at 37 °C with shaking at 180 rpm on a rotary shaker. Cells treated with DMSO were used as negative control. Finally, cells were pelleted at 3000× *g*, concentrated 10 times in fresh PBS and the hyphae formation was observed using bright field microscopy (Olympus BX51, Applied Imaging Corp., San Jose, CA, USA) under 20× magnification.

#### 3.4.4. Cytotoxicity and Adherence Assay

Cytotoxicity of the compounds was determined as antiproliferative effect on human fibroblast MRC-5 and human alveolar basal epithelial A549 cell lines (both obtained from American Type Culture Collection (ATCC)), as described previously [67].

The ability of fluorescently labeled reporter strains *C. albicans* SC5314 (GFP and RFP) to infect A549 cells was tested in adherence assay, as described previously [69]. A549 cells were grown on 22 mm glass coverslips in RPMI 1640 medium for two days. A549 monolayers on glass coverslips were inoculated with *C. albicans* SC5314 cells in log growth phase (without centrifugation) and incubated for 1 h in RPMI 1640 without fetal bovine serum (FBS) at 37 °C and 5% CO_2_. After co-incubation, nonadherent cells were removed by extensively rinsing three times with PBS and samples fixed with 4% (*v/v*) paraformaldehyde. Next, A549 cells were stained with 1 µg/mL of 2-(4-amidinophenyl)-6-indolecarbamidine dihydrochloride (DAPI, Sigma-Aldrich, Munich, Germany) for 20 min in the dark. Both A549 and *C. albicans* SC5314 cells were visualized using a fluorescence microscope (Olympus BX51, Applied Imaging Corp., San Jose, CA, USA), at a 20× magnification. 

#### 3.4.5. Ergosterol Concentration

Ergosterol levels in treated (0.25 × MIC and 0.5 × MIC concentration of fluconazole and complexes **1** and **2**) and untreated cultures of *C. albicans* ATCC 10231 were estimated according to the published procedure [72], with minor modifications. Treatments were done for 18 h at 37 °C on the rotary shaker (180 rpm). Ergosterol concentrations were determined spectrophotometrically by scanning absorbance between 240 nm and 300 nm using Ultrospec 3300pro 573 (Amersham Biosciences, Amersham, UK).

### 3.5. Molecular Docking

Molecular docking was applied for the determination of interaction between complexes **1** and **2** and target enzyme and calculation of binding energies. A semi-empirical quantum chemistry approach (PM6) was used for the optimization of appropriate geometry of the studied complexes **1** and **2**. Excellent compromise between the computational time and the description of the electronic correlation was the main reason for choosing semi-empirical (PM6) approach [73,74,75,76,77]. For all calculations related to geometry optimization, Gaussian 09 software package was applied. As a target enzyme, cytochrome P450 sterol 14α-demethylase (CYP51) from *Mycobacterium tuberculosis* in the complex with fluconazole was chosen (PDB: 1EA1). As the main software for docking studies, Molegro Virtual Docker (MVD v. 2013.6.0.1.) was employed with rigid enzyme model and flexible complexes. The visualization of the lowest energy poses was done using CHIMERA (http://www.cgl.ucsf.edu/chimera/) molecular graphics program. 

### 3.6. Antibacterial and Anti-quorum Sensing Properties of Fluconazole and Complexes ***1*** and ***2***

MIC values of complexes **1** and **2** and fluconazole were determined according to the standard [78]. Bacterial test organisms included: *Pseudomonas aeruginosa* ATCC 10332, *P. aeruginosa* PA14, *P. aeruginosa* BK25H, *P. aeruginosa* S20 [79], *Escherichia coli* NCTC 9001, *Staphylococcus aureus* ATCC 25923*, S. aureus* NCTC 6571, and *S. aureus* ATCC 43300 MRSA (methicillin-resistant *Staphylococcus aureus*). The inoculums were 5 × 10^5^ cfu/mL for all tested bacteria species.

Anti-biofilm activity of complexes was assessed on *P. aeruginosa* ATCC 10332 bacterial strain. Formed biofilm was quantified with crystal violet (CV) staining in 96-well microtiter plates by previously described methodology [80].

#### 3.6.1. *Chromobacterium violaceum* CV026 Disc Assay

*Chromobacterium violaceum* CV026 was used for the assessment of the violacein production controlled by quorum sensing [81]. This strain was cultivated overnight at 30 °C and 180 rpm. Into semi-solid LB (Luria–Bertani) agar (0.3%, *w*/*v*, 5 mL), 50 µL of an overnight culture of *C. violaceum* CV026 was seeded and supplemented with *N*-hexanoyl-l-homoserine lactone (Sigma, Munich, Germany) to a final concentration of 5 µM and it was poured over the surface of LB agar plates. After solidification, the sterile discs were placed on the surface of plates and the tested compounds were added in appropriate concentrations. Petri dishes were incubated at 30 °C in an upright position overnight. Inhibition of violacein production was defined as the presence of blurry white hallows around discs containing active compound.

#### 3.6.2. *Serratia marcescens* Disc Assay

Overnight culture of *S. marcescens* was diluted 100-fold in semi-solid LB agar (0.3%, *w*/*v*), and after poured over LB agar plates. After solidification, the sterile discs were placed into the surface of plates and the tested compounds were added in appropriate concentrations. Plates were incubated for 24 h at 30 °C in an upright position [82]. Inhibition of prodigiosin synthesis was identified by the absence of red color around the disc.

#### 3.6.3. Pyocyanin Assay

The assay used for the determination of pyocyanin production was previously published in 2013 by O’Loughlin and coworkers [83]. *P. aeruginosa* PA14 strain was cultivated overnight, and that culture was subcultured 1:1000 in 5 mL fresh LB medium. Tested complexes were assayed in a concentration of 50 µg/mL (subinhibitory concentration) and the incubation period was 20 h at 37 °C on a rotary shaker 180 rpm. OD_600_ was measured for full cultures, and cells were separated from culture fluids by centrifugation, 3000× *g* for 15 min. OD_695_ of supernatants were measured on spectrophotometer Ultrospec 3300pro 573 (Amersham Biosciences, Amersham, England). Values of OD_695_ were normalized per cell density.

#### 3.6.4. Evaluation of Quorum Sensing Inhibition Potential Using Biosensors

Overnight cultures of biosensors *P. aeruginosa* PA14-R3 (ΔlasI Prsal::lux) [84], PAOJP2/pKD-rhlA (ΔrhlA PrhlA::lux) [85] and *P. aeruginosa* PAO1 ΔpqsA (CTX lux::pqsA) [86] were diluted to 0.045 optical density measured at 600 nm (OD_600_) and incubated with complexes (50 µg/mL) in the presence of 5 μM of corresponding specific autoinducers for 4 h at 37 °C on a rotary shaker (70 rpm). OD_600_ and bioluminescence were simultaneously measured using a Tecan Infinite 200 Pro multiplate reader (Tecan Group Ltd., Männedorf, Switzerland). Luminescence values were normalized per cell density [87]. 

## 4. Conclusions

This study demonstrates that the clinically used antifungal agent fluconazole (fcz) exhibits high affinity to coordinate Cu(II) and Zn(II) ions, resulting in the formation of polymeric {[CuCl_2_(fcz)_2_]^.^5H_2_O}_n_, **1**, and {[ZnCl_2_(fcz)_2_]·2C_2_H_5_OH}_n_, **2**, complexes of octahedral geometry. A comparison of in vitro anti-*Candida* activity between these two complexes and fluconazole showed that complexation of this antifungal agent with Zn(II) ion resulted in 5.4- to 11-fold increased activity in the case of *C. parapsilosis* and *C. krusei*, respectively. Furthermore, both complexes prevented hyphae formation of *C. albicans* to a comparable level with fluconazole and showed good to moderate effect on biofilm formation of *C. albicans* and *C. parapsilosis*, with complex **1** demonstrating slightly higher efficiency in the anti-biofilm activity. Contrary to this, analysis of the effect on fluorescent *Candida* species adherence to A549 cells in the presence of the complexes showed that zinc(II) complex **2** is reducing the adhesion of fungal cells, while fluconazole and copper(II) complex **1** are not showing that effect under the tested conditions. All three compounds reduce the total amount of ergosterol at subinhibitory concentrations, with complex **1** being the most potent, while complex **2** and fluconazole show the comparable effect. Molecular docking analysis of the complexes **1** and **2** and CYP51 showed that both complexes have electrostatic, π stacking, and hydrogen bonding interactions with the enzyme amino acids. Additionally, from this study one could conclude that complex **2** has higher electrostatic and steric interactions, as well as higher internal energy, in comparison to complex **1.** Finally, complex **2** reduces pyocyanin production in *Pseudomonas aeruginosa* and inhibits its biofilm formation, suggesting that this complex could be further exploited as an agent for the treatment of the mixed *Candida-Pseudomonas aeruginosa* infections. 

## Figures and Tables

**Table 1:** Antifungal (MIC) activity of complexes **1** and **2** in comparison to fluconazole on different *Candida* species and their cytotoxicity against healthy human fibroblasts MRC-5 (IC_50_).

Tested Organisms	1		2		Fluconazole	
	µg/mL	µM	µg/mL	µM	µg/mL	µM
*C. albicans* ATCC 10231	50	59.8	3.12	3.71	0.88	2.87
*C. parapsilosis* ATCC 22019	1.75	2.09	0.88	1.05	1.75	5.71
*C. krusei* ATCC 6258	50	59.8	3.12	3.71	12.50	40.81
*C. albicans* 1c ^a^	2	2.39	2	2.38	2	6.53
*C. albicans* 1f	2	2.39	2	2.38	2	6.53
*C. albicans* 11	2	2.39	2	2.38	2	6.53
*C. albicans* 13	2	2.39	2	2.38	2	6.53
MRC-5	72 ± 2	86 ± 2	65 ± 4	77 ± 5	300 ± 8	980 ± 26

^a^*C. albicans* 1c, *C. albicans* 1f, *C. albicans* 11 and *C. albicans* 13 are clinical isolates from veterinary and human samples.

**Table 2:** Effect of complexes and fluconazole on pyocyanin production and biofilm formation in *P. aeruginosa* isolates. Tested concentration for pyocyanin assessment was 50 µg/mL, while for biofilm inhibition was 25 µg/mL.

Compounds	Pyocyanin Inhibition, %			Biofilm Inhibition, %
	*P. aeruginosa* PA14	*P. aeruginosa* DM50	*P. aeruginosa* S20	
**1**	0	0	0	
**2**	26 ± 8	25 ± 1	13 ± 3	25.2 ± 9
**fcz**	0	12 ± 10	0	

## Data Availability

The spectroscopic data used to support the findings of this study are available on request from the corresponding author.

## Supplementary Materials

The following are available online at https://www.mdpi.com/1424-8247/14/1/24/s1, Experimental data for fluconazole (fcz), Figure S1: Overlay of the metal coordination spheres in complexes **1** and **2**. Chlorido ligands bound to Cu(II) and Zn(II) ions are displayed in dark and light green, respectively, Figure S2: Hydrogen bond network in the structure of complex **1**. Hydrogen bonds are marked in blue. The hydrogen bond network acts as an additional bridging entity in the three-dimensional polymeric structure cross-linking parallel chains further adding to lattice stability, Figure S3: Hydrogen bond network in the structure of complex **2**. Hydrogen bonds are marked in blue. The hydrogen bonds provide additional weak stabilization of the crystal lattice, Figure S4: (**a**) Stability of complex **1** over time followed by UV-Vis spectrophotometry at 25 °C in DMSO; (**b**) Stability of complex **2** in DMSO-*d*_6_/D_2_O (*v*/*v* 3:1) solution over a period of 48 h followed by ^1^H NMR spectroscopy, Table S1: Selected bond distances (Å) and valence angles (°) in complexes **1** and **2**, Table S2: Details of the crystal structure determination for complexes **1** and **2**, Table S3: Effect of complex **2** and fluconazole on *P. aeruginosa* quorum sensing signaling pathway. The tested concentration was 50 µg/mL.
